# Water Supply on Active Service

**Published:** 1919-04-12

**Authors:** 


### WATER SUPPLY ON ACTIVE SERVICE.

### Purification and Distribution Problems.

To provide an adequate supply of drinking, cooking, and washing water for a large body of men is no easy matter, even at home and in piping times of peace. But on active service the difficulties are, naturally, very much increased. In the parts of France and Belgium occupied by the British armies the civil inhabitants have often had to depend on local wells for their water. In the larger towns, main supplies had been provided before the war, but some of these were practically obliterated in the wholesale destruction of the country fought over. In any case these were often quite inadequate to deal with the sudden enormous increase in population caused by the advent of an army in the area; so that, to a great extent, the question of water supplies has had to be tackled *de novo*. The provision of water is one of the duties of the Royal Engineers, but questions as to its suitability are decided by the Medical Services, and they also decide on the method to be employed for its purification, if any is required. At each Army and Corps Headquarters there is a Royal Engineer officer " in charge of water supplies." In a Division the C.R.E. is the responsible officer. A new departure during the war has been the formation of units whose sole function is the supply of water. These are called " Water Tank Companies "; they convey water, by means of mechanically driven vehicles, wherever it may be required.

A Water Tank Company consists of two parts: one for purifying water, and the other for carrying it. The water is carried in iron tanks mounted on motor chassis, and the tanks have a capacity of 150 or 500 gallons. The sterilizing lorries are capable of dealing with 400-1,200 gallons an hour. In the original type sent out to France the water was pumped into a tank, where it was filtered and sterilised by being brought into contact with bleaching powder; it was then de-chlorinated by means of other chemicals. From the sterilizer the water was pumped to the lorries or water-carts of the different units requiring it. The later types are an improvement on this, for the sterilizing is done with chlorine gas, which is stored in iron cylinders. This is a much quicker process; the amount of water passed through is increased to 800, and even 1,200, gallons an hour in the newest machines. There are also a certain number of similar lorries, called " de-poisoners," which *can* be used for sterilizing water also, but were primarily intended to free water from any poisons which might be intentionally or accidentally introduced into it. The total carrying capacity of these units is about 40,000 gallons; they were most useful in supplying water during an advance, before fixed water points could be established. Attached to each Company are three trained, analytical chemists and thirty-six N.C.O.s and men of the R.A.M.C., who supervise the purification, or de-poisoning of the water, and make the necessary tests.

Another unit concerned with water supply is the Mobile Hygiene Laboratory in charge of a R.A.M.C. officer, specially trained and experienced in the analysis of water, food, etc., with a sergeant who acts as laboratory attendant. These are motor caravans fitted up as chemical laboratories; in practice it has been found that very little work can actually be done in the van itself, and some convenient building has usually been taken over for this purpose. Samples of water, etc., are sent in to the laboratory from all parts of the area allotted to it, and reports are sent out with a full chemical and bacteriological analysis. These units, like water tank companies, were a creation of the war; and they too have proved invaluable. In addition, every regimental medical officer, field ambulance, and sanitary section is provided with one, or more, " Horrock's " water-testing cases, and a case of reagents for testing poisons. A weak solution of bleaching powder is added to the water to be tested, which is then examined for the purpose of free chlorine by a chlorimetric method.

Where navigable river or canals existed, barges were also used instead of, or in addition to, the sterilising lorries; these could turn out 4,000 gallons of sterilised water an hour, and were identical with the lorries in their method of working.

During the prolonged stages of trench warfare in Flanders it was possible to establish large central systems, which fed, by means of pipe lines, the greater part of an army area, and were carried well forward; in fact to the furthest point to which water carts could be safely taken. In one such system the water was drawn from the Yser River and was pumped into a settling tank of sailcloth, where the matter in suspension was precipitated by the addition of alum, or alumino-ferric. This process took about eight hours, and the reaction of the water at the in-take had to be closely watched. If there was an acid reaction, the precipitation would not take place without the addition of lime, or some other alkaline substance. The clarified water was then run on to a shallow, graduated sand filter, and from there to another tank where it was mixed with a solution of bleaching powder. The plant for the first two stages was duplicated so as to save time, and to enable one of the precipitation tanks to be cleared of sludge every week. The chlorinated water was then pumped into a pipe system; by this means nearly a quarter of a million gallons of water were distributed all over the area.

A similar installation was begun in Ypres, but considerable damage was done by shell-fire, and the work had to be abandoned. On the other hand, in the chalky country of the Somme the main water supply was derived from wells, supplemented by borings undertaken by the Royal Engineers, from which the water was pumped into tanks. In such a system the water is supplied untreated so that the process of purification by the addition of bleaching powder has to be done by the unit using

**Water Supply on Active Service**—(*continued*).

the water. The horse-drawn "service" water-cart is filled from the tank, and then the necessary amount of bleaching powder is added by the R.A.M.C. man attached to the cart, and, after half-an-hour's contact, the water is fit for use.

The water inspectors of Sanitary Sections make frequent visits to the water points and test the water—the result being stated on a notice board fixed to each tank, as follows:—"Drinking water — requires — measures bleaching powder per water cart." The inspectors also examine water carts as they leave the points, to make sure that the chlorination has been probably done, and that the carts are fully equipped. Each cart carries with it tins of bleaching powder, and also alum—in case clarification is also required.

The system of water-purification by chlorination has certainly been a great success during this war, but it has certain disadvantages. First, the unpleasant taste, especially if there has been any over-chlorination; this is most noticeable after boiling and makes tea an abomination. Secondly, the difficulty of keeping bleaching powder dry—for it loses its available chlorine in the presence of moisture. The French use a similar method of water purification, known as "Javelisation" from the "Extract de Javel" (Sodium Hypochlorite solution) which is added to the water. The apparatus employed is ingenious but much more complicated than our own, and involves a certain number of calculations which, in unskilled hands, may give rise to error.

In the actual trenches, water is carried up at night in petrol tins, having been previously chlorinated in water carts. Water for washing, and drinking water for horses, is usually drawn from the nearest well, and is untreated. On one occasion, a large lake near Ypres, which supplied many water points with thousands of gallons of water daily, was reported to have been polluted. This was one occasion on which the sterilizing lorries were a great help, for one was posted at each water point, and all the water was passed through them and issued sterilized to the water lorries and carts. And, during a rapid advance, an area was reached where the main water supply would probably have to be drawn from a canal in which were many barges loaded with explosives. The question asked was whether the water would be poisoned if the enemy blew up the barges before they left!

The most anxious time of all, though, was always during an advance—before there had been time to make bores, and when the only sources of water supply for the advancing troops were wells. These were often deliberately fouled by the enemy, who filled them with bodies, manure and every kind of filth, and smashed the pumps and windlasses attached to them.

